# Baseline heart rate *Z*-score is associated with early RPP trajectory during dexmedetomidine–sufentanil sedation after pediatric transcatheter closure

**DOI:** 10.3389/fped.2026.1793589

**Published:** 2026-03-16

**Authors:** Feng Li, Ling Zou

**Affiliations:** Pediatric Intensive Care Unit (PICU), Zigong First People’s Hospital, Zigong, Sichuan, China

**Keywords:** congenital heart disease, dexmedetomidine, heart rate *Z*-score, hemodynamic monitoring, pediatric, rate–pressure product, transcatheter closure

## Abstract

**Background:**

Dexmedetomidine (DEX) plus sufentanil (SF) sedation is commonly used after transcatheter closure for simple congenital heart disease (CHD), yet early hemodynamic responses vary. Absolute heart rate (HR) values are not comparable across pediatric ages; an age-standardized HR *Z*-score may better contextualize baseline HR status.

**Objective:**

To evaluate the association between baseline HR *Z*-score and early postoperative trajectories of rate–pressure product (RPP), HR, and mean arterial pressure (MAP) under a standardized DEX + SF pathway, and to describe safety outcomes.

**Methods:**

We retrospectively studied 96 children (0–16 years) admitted to the PICU after transcatheter closure (June 2023–August 2025). Hemodynamics were recorded at admission (T0), 1 h, 4 h, and 8 h. Longitudinal associations were assessed using linear mixed models including time, HR *Z*-score, and a time × *Z*-score interaction, with age as a covariate, with sensitivity analyses.

**Results:**

RPP decreased over time and showed a significant time × HR *Z*-score interaction, indicating that baseline HR *Z*-score modified the early postoperative RPP trajectory. The corresponding interaction was significant for HR but not for systolic blood pressure or MAP, consistent with HR-driven differences, while SBP/MAP interactions were not statistically significant in this cohort. Bradycardia occurred in 9/96 patients (9.4%), and no hypotension events were observed.

**Conclusion:**

Baseline HR *Z*-score, as an approximate age-standardized index of heart rate, is associated with early postoperative myocardial workload trajectory after transcatheter closure under DEX + SF sedation, primarily via HR reduction, and may help guide monitoring intensity.

## 1 Introduction

Congenital heart disease (CHD) constitutes one of the most prevalent congenital malformations, with an overall incidence of approximately 0.8% to 1%. Simple lesions, such as atrial septal defect (ASD), ventricular septal defect (VSD), and patent ductus arteriosus (PDA), account for a significant proportion of these cases (1). With advancements in interventional techniques, percutaneous transcatheter closure has become the preferred treatment for simple CHDs, offering distinct advantages including minimal invasiveness, expedited recovery, and avoidance of cardiopulmonary bypass (2, 3). However, despite the reduced surgical trauma compared to open-heart surgery, pediatric patients still encounter multiple stressors during the early postoperative period, such as environmental transitions, procedural stimulation, and separation anxiety. Furthermore, as the cardiac anatomy has just undergone structural repair, the hemodynamic system enters a phase of re-equilibrium. Excessive fluctuations in heart rate (HR) and blood pressure during this critical window may elevate myocardial oxygen consumption, induce ischemia, precipitate electrophysiological instability, or potentially compromise device stability (3, 4).

The rate-pressure product (RPP), defined as the product of HR and systolic blood pressure (SBP), is a well-established surrogate marker for myocardial workload and oxygen consumption (5–7). Studies in adult coronary artery disease and cardiac surgery have demonstrated that RPP correlates strongly with direct measurements of myocardial oxygen consumption, reflecting the frequency and pressure load on the myocardium more comprehensively than isolated HR or SBP measurements (5, 6). In the pediatric cardiac perioperative setting, RPP is frequently utilized to assess the balance between myocardial oxygen supply and demand (7, 8). For children following transcatheter closure, early postoperative hemodynamic variability driven by pain or anxiety necessitates effective monitoring. Tracking RPP trends facilitates a more accurate assessment of myocardial load, thereby guiding the optimization of sedation and analgesia strategies.

To mitigate postoperative stress and maintain hemodynamic stability, perioperative sedation protocols have become increasingly standardized. Dexmedetomidine (DEX), a highly selective alpha2-adrenergic receptor agonist, provides sedative, anxiolytic, and sympatholytic properties. It induces a sedation state resembling natural sleep typically without significant respiratory depression (9, 10). By reducing sympathetic outflow and enhancing vagal tone, DEX decreases HR and moderately lowers blood pressure, thereby attenuating RPP and myocardial oxygen demand (9, 11). Sufentanil (SF) is a potent opioid that ensures stable analgesia (12). The co-administration of DEX and SF provides adequate sedation and analgesia while suppressing postoperative sympathetic surge. This combination is widely adopted in pediatric cardiac care (11, 13, 14). However, even under identical protocols, the magnitude of hemodynamic reduction varies substantially among individuals, suggesting that baseline autonomic status and relative heart rate levels may influence the response to sedatives (11, 15).

Normal HR ranges in children vary significantly with age; for instance, the resting HR of infants may reach 120–140 bpm, whereas that of school-aged children is typically 80–100 bpm (16, 17). Consequently, assessing tachycardia or bradycardia based solely on absolute values lacks comparability across age groups. To address this, an age-standardized metric is required to quantify the deviation of an individual's heart rate relative to age-matched norms (17, 18). The *Z*-score, widely used in pediatric anthropometry, standardizes measurements using the population mean and standard deviation (SD), thereby normalizing for age and gender (18, 19). Transforming the HR at PICU admission into a *Z*-score (HR *Z*-score) provides an objective measure of the “relative magnitude” of the baseline HR, offering a unified scale to analyze its impact on hemodynamic responses (18, 19).

Currently, research on the utility of HR *Z*-scores in pediatric cardiac sedation management remains limited. Existing literature has primarily focused on general hemodynamic trends under DEX (11, 13), with few studies exploring the quantitative association between baseline HR *Z*-scores and postoperative changes in RPP, MAP, and HR. Furthermore, it remains unclear whether HR *Z*-scores correlate with safety outcomes, such as the risk of bradycardia, and whether they can serve as a metric to identify children who may exhibit profound hemodynamic shifts under a standardized protocol (18, 20, 21).

Therefore, this study aimed to introduce HR *Z*-score as an age-standardized metric in children with simple CHD undergoing transcatheter closure under a standardized DEX + SF sedation pathway. Specifically, we sought to test whether baseline HR *Z*-score modifies early postoperative trajectories of RPP and HR, and whether MAP trajectories remain comparable across baseline HR profiles.

## 2 Materials and methods

### 2.1 Study design and participants

This single-center retrospective cohort study analyzed clinical data from children with simple CHD who underwent transcatheter closure and were admitted to the pediatric intensive care unit (PICU) between June 2023 and August 2025. Inclusion criteria: (1) Age 0–16 years; (2) Diagnosis of simple CHD (ASD, VSD, or PDA) with successful percutaneous closure; (3) Tracheal extubation in the operating room immediately post-procedure, followed by transfer to the PICU for monitoring under a standardized intravenous sedation and analgesia protocol (DEX + SF). Exclusion criteria: (1) Complex CHD requiring surgical repair or concomitant extracardiac surgery; (2) Failed intervention or severe intraoperative complications (e.g., perforation, massive hemorrhage, device migration) requiring surgical conversion; (3) Preoperative severe hemodynamic instability (e.g., heart failure, shock) or requirement for postoperative mechanical ventilation, ECMO, or high-dose vasoactive support; (4) Severe hepatic/renal dysfunction or central nervous system pathology; (5) Missing key monitoring data preventing RPP or HR *Z*-score calculation. The study was approved by the Hospital Ethics Committee (No. Ethics [M] 2025-085). Given the retrospective nature of the study using de-identified data, the requirement for informed consent was waived.

### 2.2 Anesthesia and postoperative sedation protocol

All procedures were performed under general anesthesia. Induction and maintenance were managed by the attending anesthesiologist and were not stratified for this analysis. Following the procedure, extubation was performed in the operating room after confirming respiratory and circulatory stability. Upon transfer to the PICU, patients were managed according to the departmental standardized sedation and analgesia protocol: (1) Loading Dose: DEX was infused at 0.5–1.0 mcg/kg over 10–15 min, and SF at 0.1–0.3 mcg/kg (22). Doses were titrated within this range by the attending physician based on weight and recovery status. (2) Maintenance Infusion: DEX was continuously infused at 0.2–0.6 mcg/kg/h, and SF at 0.03–0.05 mcg/kg/h (22). Target sedation levels were a Ramsay score of 2–3 and a FLACC score of 0–1 (23, 24). Rates were dynamically adjusted based on clinical assessment. (3) Weaning: Infusions typically continued until the following morning, with gradual tapering. This study focused on the first 8 h; detailed subsequent adjustments were not analyzed. Rescue midazolam was administered for significant agitation, and non-opioid analgesics were used for breakthrough pain if FLACC scores increased. T0 was defined as the initial set of stable vital signs recorded upon PICU admission, prior to initiation of the DEX loading dose. These measurements were obtained with the child in a supine position, breathing spontaneously, and after initial stabilization following transfer (typically within the first 5–10 min after PICU arrival).

### 2.3 Monitoring and data collection

Continuous monitoring was performed using a multi-parameter monitor (Mindray BeneVision N15, Shenzhen, China), including ECG, heart rate (HR), non-invasive blood pressure, pulse oximetry (SpO_2_), and respiratory rate. Hemodynamic data were extracted at four standardized time points: PICU admission (T0), 1 h (T1), 4 h (T4), and 8 h (T8). The rate–pressure product (RPP) was calculated as HR × systolic blood pressure (SBP) and reported as bpm·mmHg. Adverse events were extracted from the medical record. Bradycardia was defined as a documented episode of HR below the age-specific lower limit of normal lasting ≥30 s and/or judged clinically significant by the clinical team, in accordance with institutional practice based on pediatric advanced life support guidance (25). Hypotension was defined as SBP or mean arterial pressure (MAP) > 20% below the age-specific lower limit of normal accompanied by clinical signs of hypoperfusion.

### 2.4 Calculation and grouping of HR *Z*-scores

Baseline HR was standardized using a *Z*-score approach (18, 19). Age-specific reference values (mean and range) were derived from the Pediatric Resident Handbook (16), which provides norms specifically for the Chinese pediatric population. As the handbook reports normal intervals rather than LMS parameters or full distributional statistics, the standard deviation (SD) was approximated using the formula SD ≈ (upper limit − lower limit)/4, under the assumption that the reported normal range roughly corresponds to mean ± 2 SD. The baseline HR *Z*-score was calculated as: HR *Z* = (HR(T0) − Mean_HR_age)/SD_HR. Because paediatric HR distributions may deviate from normality and the handbook “normal range” may not coincide exactly with ±2 SD, these HR *Z*-scores should be interpreted as approximate standardized indices rather than precise percentiles derived from large-scale epidemiological datasets. Patients were categorized into Low-*Z* (*Z* < −1.0), Normal-*Z* (−1.0 ≤ *Z* ≤ 1.0), and High-*Z* (*Z* > 1.0) groups. The age-specific reference values and the corresponding SD approximations used to derive HR *Z*-scores are provided in Supplementary Table S1.

### 2.5 Statistical analysis

Data were analyzed using SPSS (version 27.0). Continuous variables are presented as mean ± SD or median (IQR), and categorical variables as counts (percentages). The primary outcome was the trajectory of rate–pressure product (RPP) across four time points after PICU admission (T0, 1 h, 4 h, and 8 h), while HR, SBP, and MAP trajectories were analyzed as secondary/mechanistic outcomes. For longitudinal analyses, the dataset was restructured into long format (four observations per patient). Linear mixed models (LMMs) were fitted with time (categorical), baseline HR *Z*-score (continuous), and a time × HR *Z*-score interaction as fixed effects, with age included as a covariate. Within-subject correlation across repeated measures was modeled using an unstructured correlation (UNR) covariance matrix as the primary structure; for the RPP model, a sensitivity analysis using an AR(1) covariance structure was performed to evaluate robustness to covariance assumptions. Given the correlation between age and weight, an alternative LMM was fitted replacing age with weight as the covariate. In a further complete-case sensitivity analysis restricted to patients with documented DEX loading doses, DEX loading dose, weight, and procedure duration were included as additional covariates (*n* = 76). As an additional sensitivity analysis, HR *Z*-score was replaced with baseline HR at PICU admission (HR at T0) in the RPP mixed model (adjusted for age) to assess whether the main trajectory finding was dependent on the *Z*-score scaling approach (Supplementary Text S1). Denominator degrees of freedom were estimated using the Satterthwaite approximation (26). Two-sided *p* < 0.05 was considered statistically significant.

Sedation dosing data (DEX loading and/or maintenance, SF dosing, rescue midazolam) were not available for a small subset of patients due to incomplete documentation. These missing data were assumed to be missing at random with respect to the hemodynamic outcomes. Analyses involving sedation doses (Table 2 and sensitivity models including DEX dose) were therefore conducted as complete-case analyses, whereas all 96 patients were included in the primary hemodynamic analyses. Safety outcomes (bradycardia and hypotension) were summarized descriptively; given the small number of bradycardia events and the absence of hypotension events, formal between-group hypothesis testing for safety endpoints was not prioritized.

## 3 Results

### 3.1 Baseline characteristics

Baseline characteristics by HR *Z*-score group are summarized in Table 1. In the overall cohort, the median age was 5.0 years and the median weight was 18.4 kg. Children in the High-*Z* group tended to be older and heavier than those in the Low-*Z* group (age: 4.5 [3.0–5.0] vs. 6.0 [4.0–8.5] years; weight: 16.3 [13.8–18.0] vs. 20.1 [16.8–24.3] kg), with statistically significant differences in both age and weight among the three groups (*P* = 0.037 and *P* = 0.043 by Kruskal–Wallis test, respectively). In contrast, sex distribution and underlying diagnosis (ASD/PDA/VSD) were similar across groups (*P* = 0.435 and *P* = 0.902, respectively), and procedure time did not differ significantly among the Low-*Z*, Normal-*Z*, and High-*Z* groups (median 40.0 [25.0–57.5], 35.0 [30.0–50.0], and 45.0 [32.5–65.0] min, respectively; *P* = 0.141). As expected from the grouping definition, baseline HR at T0 increased stepwise across HR *Z*-score categories (88.0 ± 7.9 vs. 98.9 ± 8.8 vs. 110.9 ± 11.7 bpm in the Low-*Z*, Normal-*Z*, and High-*Z* groups, respectively; *P* < 0.001), and consequently baseline RPP was lowest in the Low-*Z* group and highest in the High-*Z* group (8372.8 ± 866.6 vs. 9,497.3 ± 1,003.5 vs. 10,990.3 ± 1,422.3 bpm·mmHg, *P* < 0.001). In contrast, baseline SBP and MAP at PICU admission were broadly similar among the three groups, and the differences were not statistically significant (*P* = 0.210 and *P* = 0.150, respectively).

**Table 1:** Baseline characteristics by HR *Z*-score group.

Variable	Low-*Z* (*n* = 12)	Normal-*Z* (*n* = 56)	High-*Z* (*n* = 28)	*P* value
Age (years), median (Q1–Q3)^a^	4.5 (3.0–5.0)	5.0 (4.0–6.0)	6.0 (4.0–8.5)	0.037^a^
Weight (kg), median (Q1–Q3)^a^	16.3 (13.8–18.0)	18.0 (15.2–21.8)	20.1 (16.8–24.3)	0.043^a^
Male, *n* (%)^b^	5 (41.7)	24 (42.9)	8 (28.6)	0.435^b^
Diagnosis, *n* (%)^b^				0.902^b^
ASD	7 (58.3)	34 (60.7)	19 (67.9)	
PDA	4 (33.3)	20 (35.7)	8 (28.6)	
VSD	1 (8.3)	2 (3.6)	1 (3.6)	
Procedure time (min), median^a^	40.0 (25.0–57.5)	35.0 (30.0–50.0)	45.0 (32.5–65.0)	0.141^a^
HR at T0 (bpm), mean ± SD^c^	88.0 ± 7.9	98.9 ± 8.8	110.9 ± 11.7	<0.001^c^
SBP at T0 (mmHg), mean ± SD^c^	95.2 ± 5.6	96.2 ± 7.6	99.5 ± 12.1	0.210^c^
MAP at T0 (mmHg), mean ± SD^c^	68.2 ± 3.4	70.1 ± 6.7	72.8 ± 9.8	0.150^c^
RPP at T0 (bpm·mmHg), mean ± SD^c^	8,372.8 ± 866.6	9,497.3 ± 1,003.5	10,990.3 ± 1,422.3	<0.001^c^

^a^ Median (first quartile–third quartile); *P* value from Kruskal–Wallis test.

^b^ *P* value from Chi-square test.

^c^ Mean ± SD; *P* value from one-way ANOVA.

### 3.2 Sedation and analgesia dosing across HR *Z*-score groups

Sedation and analgesia dosing under the standardized DEX + SF pathway (with clinician titration within predefined dose ranges) was broadly comparable across HR *Z*-score groups (Table 2). Documentation of the DEX loading dose was available for 77/96 patients (80.2%); among those with complete records, the mean DEX loading dose was 0.95 ± 0.14 mcg/kg in the Low-*Z* group (*n* = 9), 0.94 ± 0.18 mcg/kg in the Normal-*Z* group (*n* = 47), and 0.81 ± 0.23 mcg/kg in the High-*Z* group (*n* = 21), showing a modest between-group difference (*p* = 0.040). The mean DEX maintenance rate during the first 4 h was available for 94/96 patients (97.9%) and did not differ significantly across groups (*p* = 0.117). SF loading dose records were available for 84/96 patients (87.5%), and SF loading and maintenance doses were similar across groups (*p* = 0.277 and *p* = 0.056, respectively). Rescue midazolam was administered in 5/96 patients (5.2%), with no significant difference among groups (*p* = 0.65, Fisher–Freeman–Halton exact test). In an exploratory analysis, baseline HR *Z*-score was not significantly associated with the mean DEX maintenance rate during the first 4 h after PICU admission (Pearson *r* = −0.090, *p* = 0.389; *n* = 94), suggesting that clinicians did not systematically titrate the early maintenance infusion based on admission HR *Z*-score; although pain scores at T0 were not recorded, FLACC scores assessed at 1 h, 4 h, and 8 h showed no statistically significant differences among the Low-*Z*, Normal-*Z*, and High-*Z* groups (*p* = 0.156, *p* = 0.445, and *p* = 0.115, respectively; Kruskal–Wallis test), indicating comparable analgesia during the observation period. Missingness of dosing documentation by HR *Z*-score strata is summarized in Supplementary Table S2.

**Table 2:** Sedation and analgesia dosing during the first 4 h after PICU admission by HR *Z*-score group.

Variable	Low-*Z* (*Z* < −1.0)	Normal-*Z* (−1.0 to 1.0)	High-*Z* (*Z* > 1.0)	*P* value
DEX loading dose (mcg/kg), mean ± SD (*n*)	0.95 ± 0.14 (9)	0.94 ± 0.18 (47)	0.81 ± 0.23 (21)	0.040
DEX maintenance rate 0–4 h (mcg/kg/h)	0.25 ± 0.07 (12)	0.21 ± 0.06 (56)	0.22 ± 0.09 (26)	0.117
SF loading dose (mcg/kg), mean ± SD (*n*)	0.23 ± 0.06 (10)	0.23 ± 0.03 (49)	0.21 ± 0.08 (25)	0.277
SF maintenance rate 0–4 h (mcg/kg/h)	0.049 ± 0.008 (12)	0.046 ± 0.007 (56)	0.043 ± 0.009 (28)	0.056
Rescue midazolam use, *n*/*N* (%)	0/12 (0.0)	3/56 (5.4)	2/28 (7.1)	0.65^a^

Values in parentheses indicate the number of patients with available dosing records for that variable. DEX loading dose and SF loading dose are expressed in mcg/kg (bolus doses given over 10–15 min), whereas DEX and SF maintenance rates are expressed in mcg/kg/h (continuous infusion rates). DEX loading dose was documented in 77/96 patients and SF loading dose in 84/96 patients. DEX maintenance rate data were available for 94/96 patients (missing for 2 patients in the High-*Z* group due to incomplete recording). All available cases were included in each comparison.

^a^ *P* value from Fisher–Freeman–Halton exact test.

### 3.3 Descriptive time course of hemodynamics

**Figure 1:**
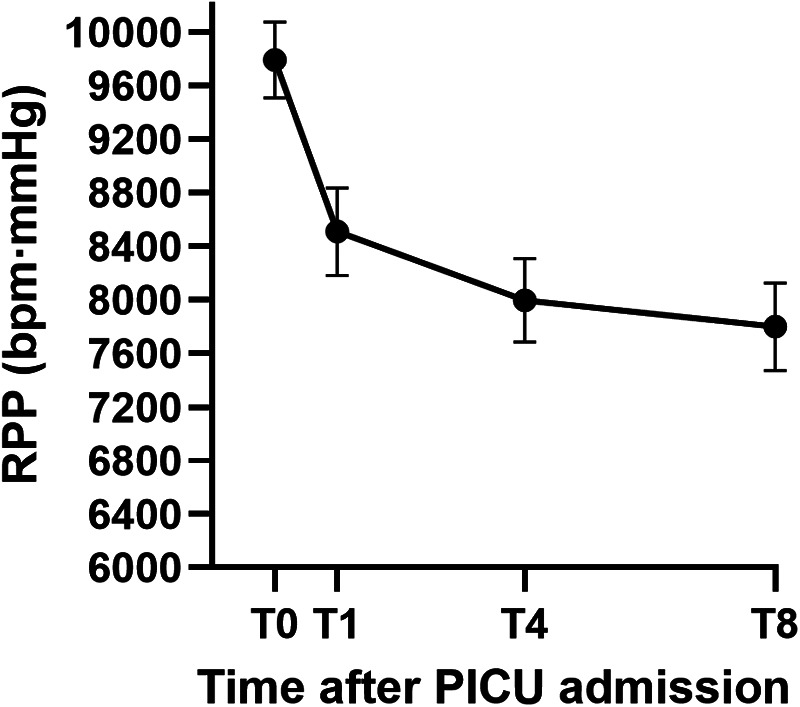
Time course of rate–pressure product (RPP) after PICU admission. Mean RPP (bpm·mmHg) at PICU admission (T0) and at 1 h (T1), 4 h (T4), and 8 h (T8) thereafter in the overall cohort (*n* = 96). Error bars represent standard deviations (SD).

During the first 8 h after PICU admission, HR decreased from 101.02 ± 12.03 bpm at T0 to 81.04 ± 15.57 bpm at T8, and RPP decreased from 9,792.20 ± 1,404.90 to 7,799.13 ± 1,610.01 bpm·mmHg, with the largest decline occurring within the first 4 h. SBP remained stable across time points (approximately 96–97 mmHg), while MAP showed a small decrease from 70.63 ± 7.54 mmHg at T0 to 67.80 ± 7.75 mmHg at T8 (Table 3; Figure 1). Correspondingly, the mean change in RPP from T0 to T4 was approximately −1,800 beats·mmHg/min, accompanied by a mean HR reduction of about 18 bpm, whereas the mean changes in SBP and MAP over the same interval were small (−1.0 mmHg [95% CI: −3.0 to 1.0] and −2.8 mmHg [95% CI: −4.5 to −1.1], respectively), indicating that the early reduction in myocardial workload was largely HR-driven rather than pressure-driven.

**Table 3:** Time course of hemodynamic parameters (overall, *n* = 96).

Time Point	HR (bpm)	SBP (mmHg)	MAP (mmHg)	RPP (bpm·mmHg)
T0 (Admission)	101.02 ± 12.03	97.06 ± 8.99	70.63 ± 7.54	9,792.20 ± 1,404.90
T1 (1 h)	87.41 ± 14.26	97.30 ± 9.05	70.59 ± 8.69	8,509.63 ± 1,617.59
T4 (4 h)	83.45 ± 15.03	96.07 ± 8.82	67.81 ± 6.82	7,996.84 ± 1,532.74
T8 (8 h)	81.04 ± 15.57	96.34 ± 8.87	67.80 ± 7.75	7,799.13 ± 1,610.01

Data are presented as mean ± SD.

### 3.4 Mixed-model trajectory analysis

**Figure 2:**
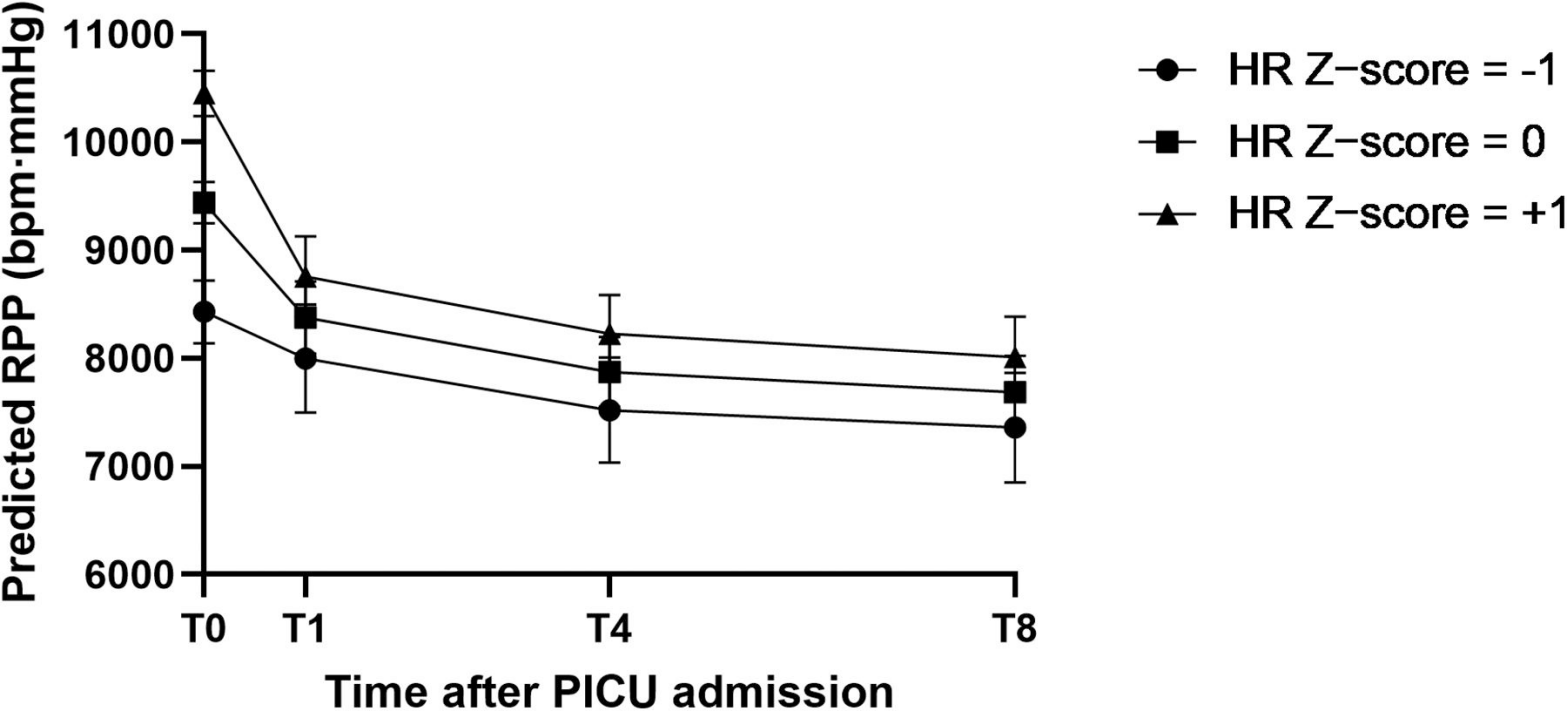
Model-predicted RPP trajectories at representative baseline HR *Z*-scores. Predicted marginal means (±95% CI) of RPP at T0, T1, T4, and T8 from the linear mixed model (UNR covariance structure), plotted at baseline HR *Z*-score values of −1, 0, and +1 (approximately −1 SD, mean, and +1 SD relative to age-specific norms), with age held at its mean (5.74 years).

Linear mixed models were used to evaluate whether baseline HR *Z*-score modified early postoperative hemodynamic trajectories under the standardized DEX + SF regimen. In the primary model for RPP adjusted for age, the time × HR *Z*-score interaction was significant, indicating that baseline HR *Z*-score modified the early postoperative trajectory of myocardial workload (Figure 2). Mechanistic decomposition showed a significant time × *Z*-score interaction for HR but not for SBP or MAP (Table 4), supporting that the differential RPP trajectory by baseline HR *Z*-score was predominantly driven by HR dynamics rather than blood pressure changes; in this cohort, the time × HR *Z*-score interactions for SBP and MAP were not statistically significant.

**Table 4:** Summary of linear mixed models for early postoperative hemodynamic trajectories (*n* = 96).

Outcome	Time effect (*F*, *p*)	Baseline HR *Z*-score effect (*F*, *p*)	Time × HR *Z*-score interaction (*F*, *p*)	Age effect (*F*, *p*)
RPP_long	48.675, <0.001	26.874, <0.001	12.829, <0.001	10.018, 0.002
HR_long	57.873, <0.001	49.593, <0.001	12.865, <0.001	526.764, <0.001
SBP_long	0.422, 0.738	0.010, 0.922	0.844, 0.473	54.769, <0.001
MAP_long	5.209, 0.002	0.005, 0.941	1.606, 0.193	39.695, <0.001

Linear mixed models adjusted for age and used an unstructured correlation (UNR) covariance matrix. The time × HR *Z*-score interaction tests whether the trajectory of each outcome over time differs by baseline HR *Z*-score. For RPP, a sensitivity analysis using an AR(1) covariance structure yielded consistent results (interaction *F* = 9.232, *p* < 0.001).

Sensitivity analyses yielded consistent results. When baseline HR *Z*-score was replaced with absolute HR at T0 in the RPP model, the time × HR(T0) interaction remained significant and the effect estimates were similar (Supplementary Text S1), indicating that the main trajectory finding did not depend on the specific *Z*-score scaling approach. In an alternative LMM in which weight replaced age as the covariate, the time × HR *Z*-score interaction for RPP remained statistically significant with similar magnitude [*F*(3, 103.8) = 12.68, *p* < 0.001], whereas weight itself was not a significant predictor of the RPP trajectory [*F*(1, 49.3) = 0.80, *p* = 0.38]. In a further complete-case sensitivity analysis restricted to patients with complete DEX dosing records (*n* = 76), including weight, DEX loading dose, and DEX maintenance dose as covariates, the time ×HR *Z*-score interaction again remained highly significant [*F*(3, 117.3) = 12.16, *p* < 0.001], while weight, DEX loading dose, and DEX maintenance dose were not significant predictors of the RPP trajectory (all *p* ≥ 0.63) (Supplementary Text S2). In all of these models, the time × HR *Z*-score interaction for RPP remained statistically significant, whereas weight and DEX dosing variables were consistently non-significant predictors.

### 3.5 Safety outcomes

Bradycardia occurred in 9 of 96 patients (9.4%). By HR *Z*-score category, bradycardia occurred in 1/12 (8.3%) patients in the Low-*Z* group, 5/56 (8.9%) in the Normal-*Z* group, and 3/28 (10.7%) in the High-*Z* group, with no significant difference among groups [*χ*^2^(2) = 0.088, *p* = 0.957]. The nadir heart rate (hr_min) in the overall cohort was 68.5 bpm (IQR 62.0–75.75; range 52–108), whereas among patients who developed bradycardia, the nadir heart rate was 55.0 bpm (IQR 55.0–57.0; range 52–59). No hypotension events were observed during the study period (0/96). In the overall cohort, the minimum MAP (map_min) was 63.3 mmHg (IQR 59.18–68.60; range 52.0–86.7), and the minimum SBP (sbp_min) was 90.0 mmHg (IQR 87.0–95.0; range 81–130). No episodes of bradycardia were documented to require pharmacologic intervention (e.g., atropine), pacing, or discontinuation of the DEX + SF regimen; events were transient and managed conservatively without special treatment.

## 4 Discussion

This study characterized early postoperative hemodynamic changes in children with simple CHD after transcatheter closure managed with a standardized DEX + SF sedation pathway and examined whether baseline age-standardized HR (HR *Z*-score) was associated with early hemodynamic trajectories. RPP declined substantially within the first 4 h after PICU admission and then showed a smaller change between 4 and 8 h, a pattern that was largely driven by HR reduction while SBP remained stable and MAP showed only modest changes. Using linear mixed models, baseline HR *Z*-score significantly modified the time course of RPP (time × *Z*-score interaction), and this interaction remained robust under an AR(1) covariance structure. Mechanistic decomposition further showed a significant time × *Z*-score interaction for HR but not for SBP or MAP, supporting that the differential RPP trajectory was predominantly driven by HR dynamics rather than blood pressure changes; in this cohort, the time × HR *Z*-score interactions for SBP and MAP were not statistically significant.

The overall temporal dynamics of RPP observed in this cohort are consistent with prior reports describing the hemodynamic effects of DEX-based sedation in pediatric cardiac patients (11, 27, 28). The early decline in RPP is consistent with sympatholysis after the onset of sedation/analgesia, typically manifesting as HR reduction with relatively preserved systolic pressure (9–11). As an indirect indicator of myocardial oxygen consumption and workload (5–7), a reduction in RPP suggests a decrease in myocardial workload. Notably, MAP remained within a clinically acceptable range in the overall cohort, and no hypotension events were observed, suggesting that within the dosing range used in this study the DEX + SF regimen reduced myocardial demand without evidence of clinically meaningful compromise in perfusion pressure. For children undergoing post-procedural hemodynamic re-equilibration, this profile may be favorable for balancing myocardial oxygen supply and demand during early recovery (11, 29).

Introducing the HR *Z*-score provided an age-standardized way to capture inter-individual differences in baseline HR status relative to age-specific norms (18). In the present cohort, children with higher baseline HR *Z*-scores tended to exhibit larger early reductions in HR and RPP under the same pathway. This difference was evident early after PICU admission and attenuated later, consistent with the significant time × *Z*-score interaction observed in mixed models. Although a higher baseline HR *Z*-score may reflect greater pre-sedation sympathetic activation or peri-admission stress, these mechanisms were not directly measured in this retrospective study and should be interpreted cautiously (9–11). Of note, the High-*Z* group received a slightly lower weight-based DEX loading dose than the Low-*Z* group, yet still demonstrated larger early reductions, indicating that baseline HR status may contribute to heterogeneity in hemodynamic response beyond dosing alone. In a sensitivity analysis replacing HR *Z*-score with absolute HR at T0, the time × baseline HR interaction remained significant and the effect estimates were similar, supporting that the main trajectory finding was not dependent on the specific *Z*-score scaling approach and that age-standardization adds some explanatory value. Taken together, the primary and sensitivity LMM results support that the association between baseline HR *Z*-score and the early postoperative RPP trajectory is robust and is unlikely to be explained by age vs. weight differences or by modest variation in DEX dosing within the standardized pathway.

Regarding safety, bradycardia occurred in 9.4% of patients, while no hypotension events were observed. The nadir HR among bradycardia cases was 55.0 bpm (IQR 55.0–57.0; range 52–59), and no episodes required pharmacologic intervention, pacing, discontinuation of the DEX + SF regimen, or other special treatment. Given the low event count, the study was not powered to evaluate predictors of rare adverse events; therefore, whether HR *Z*-score can help identify children at higher risk of clinically significant bradycardia or low cardiac output warrants confirmation in larger prospective cohorts.

## 5 Limitations

Our study has several limitations. First, this was a single-center retrospective analysis with a modest sample size, and selection bias cannot be excluded; therefore, external validity should be confirmed in larger, multicenter cohorts. Second, HR *Z*-scores were derived from age-specific reference ranges in the Pediatric Resident Handbook (China), with standard deviations approximated from normal ranges because LMS parameters are not available for pediatric resting HR, particularly for the Chinese population. This approach assumes that the reported normal range corresponds approximately to mean ± 2 SD and that HR distributions are roughly normal within each age stratum. In reality, paediatric HR distributions may deviate from normality, the handbook “normal range” may not correspond exactly to ±2 SD, and wider age strata may introduce additional estimation error for the SD. Although major international references [e.g., Fleming et al. (17) and Bonafide et al. (30)] also mainly provide percentile ranges rather than LMS-based parameters, our HR *Z*-scores should be interpreted as approximate age-standardized indices rather than precise population percentiles. In future work, the use of LMS-based reference curves or *Z*-scores derived from large-scale epidemiological data would be desirable to refine age-standardized HR assessment. Third, the sedation pathway allowed clinician titration within predefined ranges, and documentation of loading doses was incomplete for a subset of patients, which limited our ability to fully adjust for dosing in all analyses; residual confounding by indication cannot be ruled out. However, in complete-case sensitivity models including both DEX maintenance and loading doses as covariates, the interaction between HR *Z*-score (or HR at T0) and time remained significant, whereas the DEX loading dose itself was not a significant predictor of RPP trajectory. Fourth, RPP is an established surrogate of myocardial workload (5–7), but we did not have concurrent biochemical markers (e.g., lactate, troponin), echocardiographic data, or direct measures of autonomic tone to validate mechanistic interpretations. Fifth, baseline measurements (T0) were obtained early after extubation and transfer to the PICU; residual anesthetic effects, peri-admission stress, or unmeasured factors (e.g., temperature, volume status) may have influenced baseline HR and blood pressure despite similar FLACC scores across groups. Finally, adverse events were infrequent (bradycardia in 9.4% and no hypotension events), limiting statistical power to identify predictors of rare safety outcomes. Prospective studies with standardized dosing documentation, richer perioperative covariates, and multimodal monitoring are warranted to refine and validate this age-standardized risk stratification approach.

## 6 Conclusion

Under a standardized DEX + SF sedation and analgesia pathway after transcatheter closure of simple CHD in children, myocardial workload as reflected by RPP decreased substantially during the first 4 h after PICU admission and then showed a smaller change thereafter, while perfusion pressure was preserved and no hypotension events were observed. Baseline HR *Z*-score was associated with the early postoperative RPP trajectory, and mechanistic analyses suggested that this effect was primarily driven by HR dynamics rather than changes in SBP or MAP. HR *Z*-score, as an approximate age-standardized index of HR in this study, may serve as a practical metric to help anticipate which children may experience larger early reductions in HR and RPP under this regimen, potentially informing monitoring intensity. Future multicenter prospective studies with standardized perioperative data capture and broader patient populations are needed to validate generalizability and to explore whether combining age-standardized HR metrics with hemodynamic indices can support pragmatic bedside risk stratification in pediatric cardiac peri-procedural care.

## Data Availability

The original contributions presented in the study are included in the article/Supplementary Material, further inquiries can be directed to the corresponding author/s.
