# Supplementary material for: Baseline heart rate Z-score is associated with early RPP trajectory during dexmedetomidine–sufentanil sedation after pediatric transcatheter closure
Source: Front Pediatr. 2026 Mar 16;14:1793589. doi: 10.3389/fped.2026.1793589 (PMC13033676; doi:10.3389/fped.2026.1793589)
Supplement: Supplementary file 1 [file Supplementaryfile1.docx]

**Supplementary Material**

Title: Baseline Heart Rate Z-Score Is Associated With Early RPP Trajectory During Dexmedetomidine–Sufentanil Sedation After Pediatric Transcatheter Closure

Authors: Feng Li; Ling Zou

**Supplementary Table S1. Reference values used for HR Z-score calculation**

Description: Age-specific reference ranges (mean and normal range) were extracted from Li & Shen (eds.), Pediatric Resident Handbook (China, 2011). Because LMS parameters were unavailable, SD was approximated as (upper − lower)/4. HR Z-score at PICU admission (T0) was calculated as (HR(T0) − Mean)/SD. Age category assignment followed the handbook’s lower-bound cutoffs; for patients aged 12–14 years, sex-specific references were applied.For age assignment, each patient was mapped to the nearest lower-bound cutoff category in the handbook (e.g., ≥4 months, ≥7 months, ≥1 year), and sex-specific categories were applied for ages 12–14 years.

| **Age category** | **Mean HR (bpm)** | **Lower (bpm)** | **Upper (bpm)** | **Estimated SD ((Upper−Lower)/4)** |
| --- | --- | --- | --- | --- |
| Birth (reference point) | 127.9 | 88 | 158 | 17.50 |
| ≥2 days (reference point) | 116.5 | 85 | 162 | 19.25 |
| ≥8 days (reference point) | 146.0 | 115 | 172 | 14.25 |
| ≥1 month (reference point) | 139.5 | 111 | 167 | 14.00 |
| ≥4 months (reference point) | 130.0 | 105 | 158 | 13.25 |
| ≥7 months (reference point) | 124.8 | 109 | 154 | 11.25 |
| ≥1 year (reference point) | 119.2 | 85 | 187 | 25.50 |
| ≥3 years (reference point) | 108.8 | 75 | 133 | 14.50 |
| ≥4 years (reference point) | 100.8 | 71 | 133 | 15.50 |
| ≥6 years (reference point) | 91.7 | 68 | 125 | 14.25 |
| ≥8 years (reference point) | 88.9 | 64 | 123 | 14.75 |
| ≥11 years (reference point) | 82.3 | 52 | 115 | 15.75 |
| Boys 12–14 years | 77.4 | 58 | 102 | 11.00 |
| Girls 12–14 years | 87.3 | 55 | 109 | 13.50 |

Reference: Li Z, Shen K, editors. Pediatric Resident Handbook [Er Ke Zhu Yuan Yi Shi Shou Ce]. Beijing: Peking Union Medical College Press (2011). p. 6. [in Chinese].

**Supplementary Table S2. Missingness of dosing documentation by HR Z-score strata**

Description: Missingness indicators were defined as 1 if the corresponding dosing variable was not documented and 0 otherwise. Values are shown as missing and available counts with percentages calculated within each HR Z-score stratum (Z_group).

| **Z_group** | **DEX load missing n(%)** | **DEX load available n(%)** | **SF load missing n(%)** | **SF load available n(%)** | **DEX maint missing n(%)** | **DEX maint available n(%)** | **SF maint missing n(%)** | **SF maint available n(%)** |
| --- | --- | --- | --- | --- | --- | --- | --- | --- |
| 1 (Low-Z), n=12 | 3 (25.0) | 9 (75.0) | 2 (16.7) | 10 (83.3) | 0 (0.0) | 12 (100.0) | 0 (0.0) | 12 (100.0) |
| 2 (Normal-Z), n=56 | 9 (16.1) | 47 (83.9) | 7 (12.5) | 49 (87.5) | 0 (0.0) | 56 (100.0) | 0 (0.0) | 56 (100.0) |
| 3 (High-Z), n=28 | 7 (25.0) | 21 (75.0) | 3 (10.7) | 25 (89.3) | 2 (7.1) | 26 (92.9) | 0 (0.0) | 28 (100.0) |
| **Total, n=96** | **19 (19.8)** | **77 (80.2)** | **12 (12.5)** | **84 (87.5)** | **2 (2.1)** | **94 (97.9)** | **0 (0.0)** | **96 (100.0)** |

Note: DEX load = dexmedetomidine loading dose; SF load = sufentanil loading dose; DEX maint = dexmedetomidine maintenance dose (0–4 h); SF maint = sufentanil maintenance dose (0–4 h). Percentages are calculated within each HR Z-score stratum. All hemodynamic variables (HR/SBP/MAP/RPP at T0, 1 h, 4 h, and 8 h) and key covariates (Z_score, age, sex_code) were complete (0 missing).

**Supplementary Text S1. Sensitivity analysis using baseline HR at T0**

To evaluate whether the primary RPP trajectory finding depended on the Z-score scaling approach, we repeated the RPP linear mixed model by replacing HR Z-score with absolute baseline HR at PICU admission (HR at T0), while retaining the same model structure and adjusting for age. Using an unstructured correlation matrix (UN), the time × HR(T0) interaction was statistically significant (F = 6.449, p = 0.001). Results were consistent when an AR(1) covariance structure was used (F = 5.669, p = 0.001). These findings support that the observed modification of the early postoperative RPP trajectory was not dependent on the specific HR Z-score calculation method and that age-standardization, while helpful, is not the sole driver of the association.

**Supplementary Text S2. Dose-adjusted complete-case sensitivity analysis**To assess whether the observed association between baseline HR Z-score and the RPP trajectory could be explained by differences in DEX dosing, we performed a complete-case sensitivity analysis in the subset of patients with fully documented DEX loading and maintenance doses (n = 76). In this LMM, weight, DEX loading dose (mcg/kg), and DEX maintenance dose (mcg/kg/h) were added as covariates, in addition to time, HR Z-score, and the time × HR Z-score interaction, using an unstructured covariance structure and a random intercept for each patient.

In this model, the time × HR Z-score interaction for RPP remained highly significant (F(3, 117.3) = 12.16, p < 0.001), indicating that baseline HR Z-score continued to modify the early postoperative RPP trajectory. In contrast, weight, DEX loading dose, and DEX maintenance dose were not significant predictors of the RPP trajectory (F(1, 388.2) = 0.00, p = 0.995; F(1, 388.2) = 0.23, p = 0.631; and F(1, 388.1) = 0.01, p = 0.905, respectively). Although the UN covariance structure produced a non-convergence warning in this sensitivity model, the fixed-effect estimates and F-tests for the time × HR Z-score interaction were very similar to those from the primary age-adjusted models. Taken together, these results suggest that the primary RPP trajectory finding is robust and is unlikely to be driven by modest group differences in DEX dosing.

**Supplementary Text S3. Effect sizes and 95% confidence intervals for ΔSBP and ΔMAP (0–4 h)**

To further quantify the magnitude of blood pressure changes underpinning the “HR-driven” interpretation, we calculated the mean changes in SBP and MAP from T0 to T4 (ΔSBP 0–4 h and ΔMAP 0–4 h) and their 95% confidence intervals in the overall cohort (n = 96). The mean ΔSBP (0–4 h) was −1.0 mmHg (95% CI: −3.0 to 1.0), and the mean ΔMAP (0–4 h) was −2.8 mmHg (95% CI: −4.5 to −1.1). These changes were small compared with the mean HR reduction of approximately 18 bpm and the corresponding RPP decrease of about 1800 beats·mmHg/min. Together with the non-significant time × HR Z-score interactions for SBP and MAP in the mixed models, these numerical effect sizes support the conclusion that the observed differences in RPP trajectories were primarily driven by HR dynamics rather than by large shifts in arterial pressure.
